# Neglected arboviruses in South America: clinical manifestations and diagnostic challenges

**DOI:** 10.1093/trstmh/trag095

**Published:** 2026-08-27

**Authors:** Milagros Saavedra-Samillán, Sharon Díaz Velásquez, Marcos A Castillo Zuta, Julio Sandoval-Bances, Rafael Tapia-Limonchi, Stella M Chenet

**Affiliations:** Escuela de Posgrado (EPG), Universidad Nacional Toribio Rodríguez de Mendoza de Amazonas (UNTRM), 01001,Chachapoyas, Peru; Instituto de Investigación en Enfermedades Tropicales (IET), Universidad Nacional Toribio Rodríguez de Mendoza de Amazonas (UNTRM), 01001,Chachapoyas, Peru; Escuela de Posgrado (EPG), Universidad Nacional Toribio Rodríguez de Mendoza de Amazonas (UNTRM), 01001,Chachapoyas, Peru; Red Integrada de Salud Chachapoyas (RIS), Dirección Regional de Salud Amazonas (DIRESA), 01001,Chachapoyas, Peru; Instituto de Investigación en Enfermedades Tropicales (IET), Universidad Nacional Toribio Rodríguez de Mendoza de Amazonas (UNTRM), 01001,Chachapoyas, Peru; Facultad de Medicina (FAMED), Universidad Nacional Toribio Rodríguez de Mendoza de Amazonas, UNTRM, 01001,Chachapoyas, Peru; Instituto de Investigación en Enfermedades Tropicales (IET), Universidad Nacional Toribio Rodríguez de Mendoza de Amazonas (UNTRM), 01001,Chachapoyas, Peru; Facultad de Medicina (FAMED), Universidad Nacional Toribio Rodríguez de Mendoza de Amazonas, UNTRM, 01001,Chachapoyas, Peru; Instituto de Investigación en Enfermedades Tropicales (IET), Universidad Nacional Toribio Rodríguez de Mendoza de Amazonas (UNTRM), 01001,Chachapoyas, Peru; Facultad de Medicina (FAMED), Universidad Nacional Toribio Rodríguez de Mendoza de Amazonas, UNTRM, 01001,Chachapoyas, Peru; Instituto de Investigación en Enfermedades Tropicales (IET), Universidad Nacional Toribio Rodríguez de Mendoza de Amazonas (UNTRM), 01001,Chachapoyas, Peru; Facultad de Medicina (FAMED), Universidad Nacional Toribio Rodríguez de Mendoza de Amazonas, UNTRM, 01001,Chachapoyas, Peru

**Keywords:** emerging viruses, dengue, Oropouche, Mayaro, Saint Louis Encephalitis, cross-reaction

## Abstract

In South America, the arboviruses that are most noted are those that lead to dengue, Zika, and chikungunya. Nevertheless, there are additional arboviruses that trigger outbreaks, yet do not receive adequate attention, including Oropouche virus (OROV), Mayaro virus (MAYV), Saint Louis Encephalitis virus, Madariaga virus (MADV), and Rocío virus (ROCV). A rapid review was conducted using the databases PubMed, LILACS, Scopus, and Google Scholar. This analysis focused on studies that outline the clinical signs and diagnostic methods of neglected arboviruses impacting human populations in South America. A total of 38 studies met the inclusion criteria. Most reports focused on OROV and MAYV, whereas only a few studies described MADV and ROCV. Brazil accounted for the largest proportion of studies, followed by Peru. Across the reviewed literature, these arboviruses were associated with a broad clinical spectrum, ranging from non-specific febrile illness to severe neurological disease and congenital complications. Diagnostic approaches included serological assays, viral isolation or neutralization tests, and molecular methods such as RT-PCR. The increasing spread of these under-reported arboviruses in South America requires strengthening monitoring systems, optimizing access to specific diagnostics, and training healthcare personnel to identify these infections early and manage them appropriately.

## Introduction

Arboviruses, which are viruses transmitted by arthropods, have caused various diseases in humans over time.^1^ In South America, the most reported arboviruses are dengue, Zika, and chikungunya virus (CHIKV), all belonging to the family Flaviviridae.^2^ However, other arboviruses are present in this region that, although they have the potential to cause outbreaks and serious diseases, often go unnoticed. These viruses, classified as neglected viruses by Rodriguez-Morales and Bonilla-Aldana, include Oropouche virus (OROV), Mayaro virus (MAYV), Madariaga virus (MADV), Saint Louis Encephalitis virus (SLEV), and Rocío virus (ROCV).^3^

In most South American countries, these arboviruses are usually excluded from standard monitoring systems, their clinical manifestations are often non-specific and overlap considerably with other known febrile diseases, making clinical diagnosis difficult.^4^ This challenge is intensified by the unavailability of specific and affordable diagnostic tools, especially in areas with limited resources, which hampers timely detection and response efforts.

We hypothesized that neglected arboviruses in South America are characterized by overlapping clinical manifestations and diagnostic limitations that contribute to under-recognition and knowledge gaps. Therefore, this review aimed to synthesize available evidence on the clinical manifestations and diagnostic methods to inform future research and surveillance efforts.

## Methods

The search strategy was developed in January 2026, implemented in February 2026, and completed in early March 2026. Articles published from January 2005 to February 2026 were identified through searches conducted in PubMed, LILACS, Scopus, and Google Scholar. The strategy incorporated controlled vocabulary descriptors (MeSH), as detailed in the supplementary tables (Supplementary Table 1). Gray literature publications were not included in the search. Original research articles describing clinical manifestations and/or diagnostic approaches related to neglected arboviruses in human populations residing in South America were included. Reviews, editorials, and conference abstracts were excluded unless they provided primary data or detailed diagnostic methodologies.

The search results were exported to the Rayyan web application, where duplicate records were first removed. Subsequently, in the study selection stage, titles and abstracts were reviewed, followed by full-text evaluation of potentially relevant articles to determine their eligibility. Discrepancies were resolved by consensus. The study selection process is detailed in the PRISMA flowchart shown in Supplementary Figure 1. The reviewers extracted the data to an Excel spreadsheet using a standardized form; subsequently, a third reviewer verified the consistency and completeness of the information. The following information was collected: author, year of publication, population, country, description of clinical manifestations, description of diagnostic tools, and unique findings.

Methodological quality and risk of bias of the included studies were assessed using Joanna Briggs Institute (JBI) Critical Appraisal Tools. The checklist applied depended on the study design, including case reports, case series, cohort studies, diagnostic studies, analytical cross-sectional, and prevalence studies. The overall risk of bias was classified as low, moderate, or high (see Supplementary Table 2). The quality assessment was used to support the interpretation of the findings, and no studies were excluded based solely on their methodological quality.

## Results

### Characteristics of the included studies

A total of 38 articles met the eligibility criteria. Risk of bias assessment indicated that most included studies had a low to moderate methodological bias, while only one study was classified as having a high risk of bias.

In recent years, the number of articles on neglected arboviruses has been increasing (Fig. 1). In the included studies, OROV was the most frequently reported arbovirus (14/38, 36.8%), followed by MAYV (13/38, 34.2%) and SLEV (6/38; 15.8%). By contrast, only a few studies reported ROCV (2/38, 5.3%) and MADV (1/38, 2.6%). Additionally, two studies investigated more than one virus (2/38; 5.3%).

**Figure 1:**
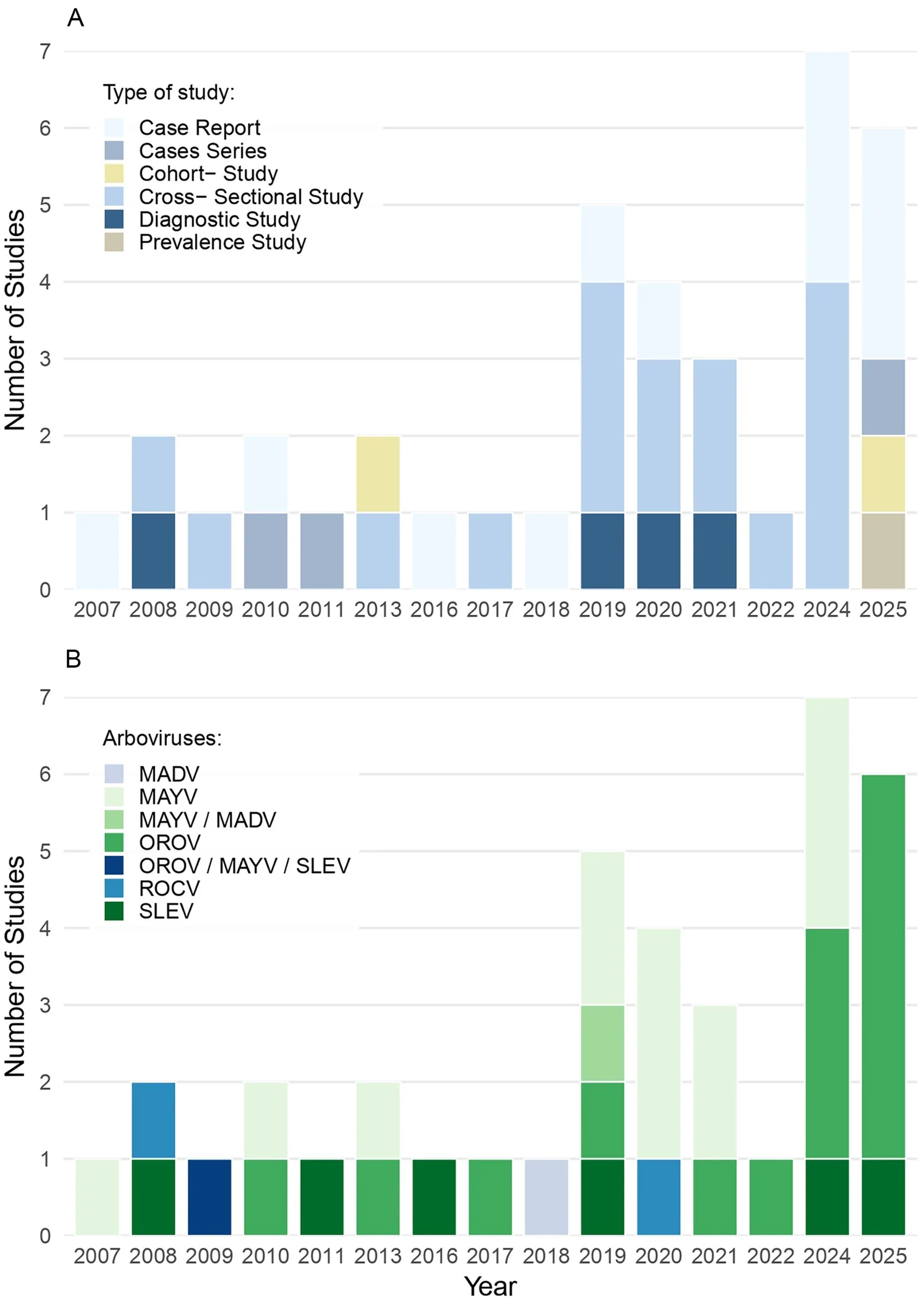
Temporal distribution of included studies by neglected arbovirus in South America (2005–2026).

In the geographic distribution analysis, Brazil accounted for the highest number of studies (15/38, 39.5%), followed by Peru (8/38, 21.1%), Argentina and Colombia (4/38 each, 10.5%), then Bolivia, Ecuador, Paraguay, Suriname, and Venezuela with one study each (1/38 each, 2.6%) (Fig. 2). Furthermore, two studies were multinational and contributed data from more than one country (2/38; 5.3%) (Fig. 2).

**Figure 2:**
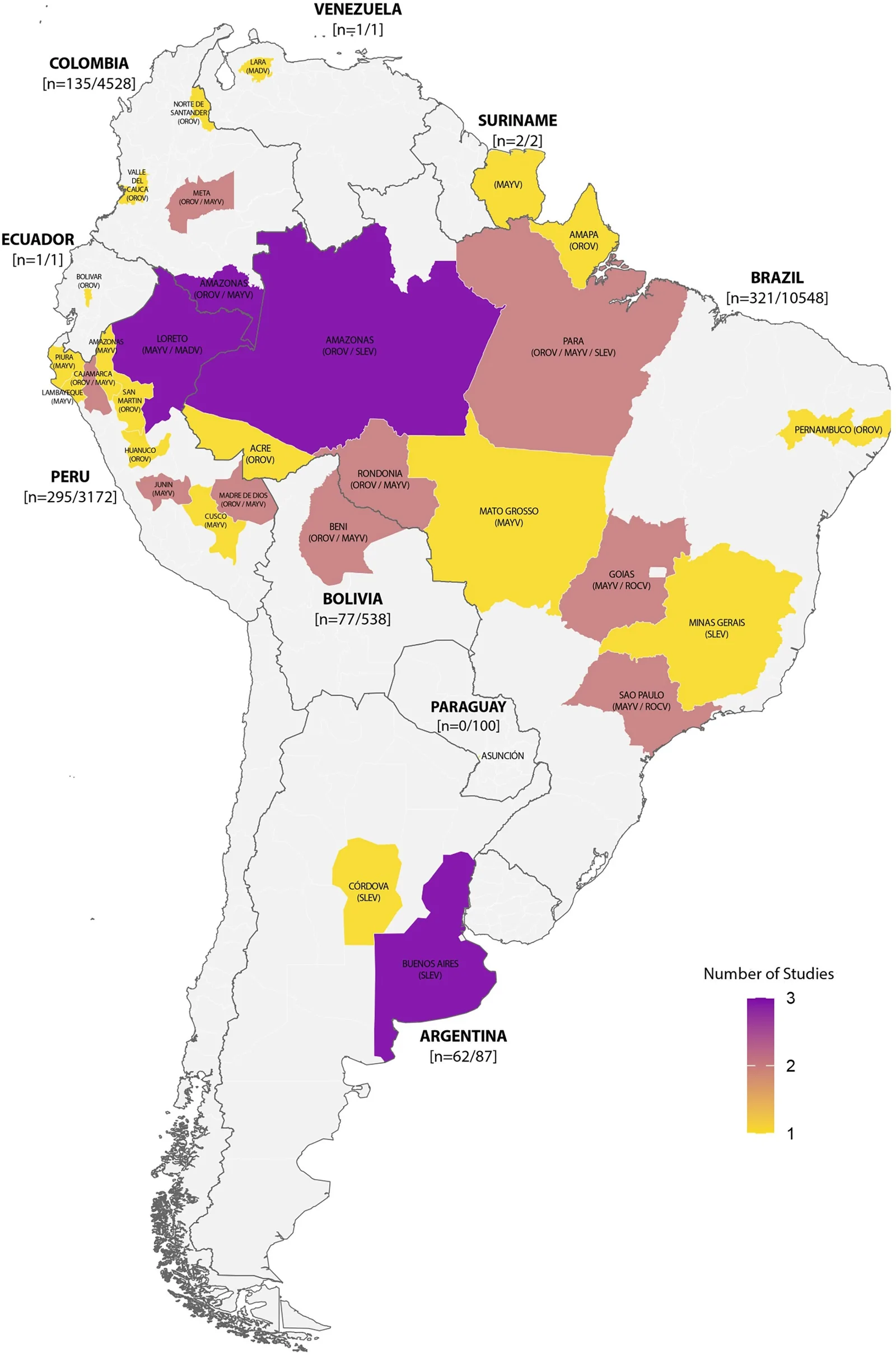
Heatmap showing the geographic distribution of neglected arbovirus studies in South America at the subnational level. The values [n] represent the total number of positive cases over samples evaluated in each country. Detailed study-level information is available in Supplementary Table 3.

### General clinical features and complications

The results of the rapid review highlight the diversity of arboviral viruses associated with febrile and neurological syndromes in different endemic regions of South America. Based on the selected articles, multiple reports were identified that describe the circulation, clinical manifestations, and detection methods for these arboviruses. The information gathered allowed us to characterize the main epidemiological and diagnostic findings for each of them (OROV, MAYV, SLEV, ROCV, and MADV). The following sections provide an individual description of each virus identified in the review, summarizing the most relevant aspects reported.

#### Oropouche virus

OROV is an arbovirus classified within the genus *Orthobunyavirus* of the family Peribunyaviridae.^5^ Transmission to humans occurs primarily through the bite of genus *Culicoides*.^6^

The most common reported clinical manifestations include fever, moderate to severe headaches, retro-orbital pain, dizziness, chills, and loss of appetite.^6–8^ Musculoskeletal symptoms frequently reported include myalgia, arthralgia, and back/lumbar pain.^7–9^ Gastrointestinal manifestations such as nausea, vomiting, abdominal pain, and diarrhea are also commonly described.^10^,^11^ Additionally, dermatological and other symptoms include exanthema or diffuse rash, and odynophagia.^12–15^

In addition to the most common symptoms, less frequent manifestations have also been reported in patients with OROV infection. These include conjunctival injection, cough, asthenia, rhinorrhea, photophobia, anorexia,^10^ chest pain, bone pain, and low platelet count.^12^ Likewise, some rare events such as hematemesis have been reported.^12^ Although these findings are reported less frequently, they highlight the wide range of clinical manifestations associated with OROV infection.

Furthermore, it has been shown that the symptoms of OROV infection can sometimes be severe, especially when the virus is transmitted from mother to child.^15^ Evidence of probable vertical transmission has been reported, as viral RNA has been detected in multiple fetal tissues.^15^,^16^ Likewise, adverse pregnancy outcomes, such as spontaneous abortions and intrauterine fetal death, have been reported.^15^ In reported cases of congenital infection, affected newborns have presented with severe neurological and developmental abnormalities, including microcephaly, ventriculomegaly, arthrogryposis, hypotonia, seizures, and feeding difficulties.^16^

Finally, for diagnostic purposes, a variety of serological and molecular tests have been shown to be available in South America. Serological assays include IgM/IgG ELISA tests (on serum and cerebrospinal fluid samples) and hemagglutination inhibition (HI).^10^,^15^,^17^ Molecular methods including RT-qPCR, conventional PCR, and the development of new techniques such as digital droplet PCR (ddPCR), which have detected OROV, provide sensitivity and specificity.^7^,^9^,^18^ Likewise, methods such as viral isolation in Vero and C6/36 cells, as well as neutralization assays such as the plaque-reduction neutralization test (PRNT), have been used.^14^,^19^

#### Mayaro virus

MAYV belongs to the genus *Alphavirus* within the family Togaviridae and is closely related to other viruses such as CHIKV, O’nyongnyong, and Ross River, among others.^20^ This virus is transmitted primarily through the bite of insects of the genus *Haemagogus* spp. found in forested areas.^21^

In general, infection with this arbovirus presents as a febrile syndrome characterized by persistent arthralgia and rash.^22^ Nevertheless, it is important to note that a study conducted in Brazil reported arthralgia in only one patient (1/49; 2.04%). Other non-specific symptoms such as headache and myalgia have been reported,^23^ as well as nausea, vomiting, and retro-ocular pain.^24^,^25^ Some studies have also described pain in the back or lower back,^26–29^ and joint involvement primarily affecting the hands, knees, and ankles.^30^

In addition to the most frequently reported symptoms, several less commonly described manifestations were reported in individual case reports or small case series, including blurred vision,^23^ asthenia, chills, jaundice, dyspnea, abdominal pain, respiratory distress, retro-orbital/ocular pain,^24^,^25^ diarrhea, weakness, and exanthema.^29^

Serological testing includes MAC-ELISA and IgM/IgG ELISA for the detection of anti-MAYV antibodies, as well as immunofluorescence assays^22^,^31^ and HI.^30^ Virus isolation and neutralization assays include PRNT50 in Vero cells,^29^,^32^ as well as viral isolation in newborn mice and C6/36 mosquito cells.^30^ Molecular detection is performed using RT-qPCR targeting viral genomic regions such as NSP1 and partial regions of the E1 and E2 envelope glycoproteins.^25^,^27^,^28^

#### Saint Louis Encephalitis virus

SLEV belongs to the genus *Flavivirus* of the family Flaviviridae that is closely related to the *Japanese encephalitis* serocomplex viruses.^33^ Transmission occurs primarily through mosquito bites, with *Culex* species acting as the principal vectors.^34^

SLEV infection typically presents as a febrile illness characterized by fever, headache, muscle pain, and general malaise^35^,^36^ and, occasionally, difficulty with oral intake, lower back pain, moderate dehydration, and other symptoms.^37^ However, neurological involvement has been described more frequently, which may include outcomes such as meningitis or encephalitis, temporal-spatial disorientation, confusion, photophobia, neck stiffness, language disorders (aphasia, palilalia, and paraphasia), tremors, myoclonus, ataxia, behavioral changes, and altered levels of consciousness.^37^,^38^ Recently, a patient was even reported to have developed opsoclonus-myoclonus-ataxia syndrome following resolution of the febrile phase.^36^

Based on diagnostic findings, brain magnetic resonance imaging (MRI) has been frequently used in the cases described.^35^,^37^,^38^ Neuroimaging abnormalities include leptomeningeal enhancement and bilateral hyperintensities of the uncus and amygdala on T2/FLAIR sequences,^38^ symmetrical thalamic lesions appearing hypointense on T1 and hyperintense on T2/FLAIR sequences,^37^ and inflammatory lesions affecting the cerebral white matter and the cervical/thoracic spinal cord.^35^

On the other hand, serum and cerebrospinal fluid samples have been used to detect specific IgM against SLEV using the mass enzyme-linked immunosorbent assay (MAC-ELISA) technique.^35^,^38^ Furthermore, as a confirmatory test, some authors have used the PRNT on paired serum samples, evaluating seroconversion.^38^,^39^ Finally, molecular techniques such as RT-qPCR targeting the 5′UTR region and nested multiplex RT-PCR targeting the NS5 gene have been employed for the detection of this virus.^39^

#### Rocio virus

ROCV is a flavivirus belonging to the genus *Flavivirus* of the family Flaviviridae. It is transmitted by mosquitoes, primarily *Psorophora ferox*, through a mosquito-bird-mosquito transmission cycle, in which birds act as reservoirs and amplifying hosts.^40^ This review identified two reviews on ROCV, one of which reported the clinical manifestations of two infected patients.

These clinical manifestations were characterized by a variety of symptoms including fever, myalgia, and arthralgia in both patients; one patient presented with prostration, abdominal pain, diarrhea, and thrombocytopenia, while the other experienced headache, retro-orbital pain, pruritus, nausea, and leukopenia.^41^ In addition, the molecular diagnostic methods reported by the authors included RT-PCR targeting the E and NS5 genes, as well as nested PCR targeting the NS5 gene.^41^,^42^

#### Madariaga virus

Only two articles describing human infection with MADV met the inclusion criteria. The clinical manifestations described included a sudden high fever (39°C–40°C), asthenia, general malaise, headache, nausea, and a maculopapular rash with intense itching that lasted ∼5 days, without arthralgia or myalgia.^43^ Diagnostic confirmation was achieved using molecular and serological methods. PCR methods for alphaviruses were employed, which allowed for the identification of MADV RNA.^43^ In addition, serological tests included PRNT80, as well as an IgG capture ELISA for antibodies against alphaviruses.^44^

In general, the wide clinical spectrum presented by these neglected arboviruses has been demonstrated. These manifestations range from non-specific febrile syndromes to severe neurological diseases or even congenital syndromes. Furthermore, these clinical manifestations often resemble those of better-known infections, such as dengue or Zika, which makes clinical recognition difficult. Therefore, the following is a summary of the most common clinical manifestations and diagnostic tools for each of the viruses of interest in this study (Table 1).

**Table 1:** Clinical manifestations, complications, and diagnostic tools of selected neglected arboviruses in South America.

Arbovirus	Clinical manifestation	Diagnostic tools
OROV	Most commonly reported clinical manifestations include moderate or severe headaches, fever, retro-orbital pain, myalgia,^6^,^7^ arthralgia with generalized joint tenderness,^10^ chills, back/lumbar pain,^8^ dizziness, diarrhea,^11^ exanthema, diffuse rash (head, torso, and limbs),^15^,^16^ odynophagia, loss of appetite.^13^,^14^ Gastrointestinal symptoms such as nausea, abdominal pain, and vomiting were also frequent.^11^,^12^**Complications:**- Hemorrhagic manifestations; epistaxis, gingival bleeding, menorrhagia.^11^	*Serological testing*: IgM/IgG ELISA in serum and/or cerebrospinal fluid^11^ and HI test.^16^,^18^*Viral isolation*: in Vero and C6/36 cell and neutralization assays included PRNT.^15^,^20^*Molecular testing* using RT-qPCR (targeting the nucleoprotein (N) gene, S, L, and M segments),^14^,^25^,^45^ conventional PCR (S, M, L segments).^6^,^7^,^10^
MAYV	Most commonly reported manifestations include persistent arthralgia, rash, fever,^22^ headache, myalgia,^23^ nausea, vomiting, retro-orbital/ocular pain,^24^,^25^, back/lumbar pain^26–29^; joint pain in hands, knees, and ankles.^30^**Complications:**- In severe cases, patients had abdominal pain, gingival bleeding, increased hematocrit, and hepatomegaly.^26^	*Serological testing*: MAC-ELISA and IgM/IgG ELISA for detection of anti-MAYV antibodies, as well as immunofluorescence assays,^22^,^31^ and HI.^30^*Viral isolation*: in newborn mice and C6/36 mosquito cells,^30^ and neutralization assays included PRNT50 in Vero Cells.^29^,^32^*Molecular testing*: RT-qPCR targeting NSP-1 and partial regions E1 and E2 envelope glycoproteins.^23–28^
SLEV	Headache, myalgia, malaise, fever,^35^,^36^ difficulty with oral intake, lumbar pain, behavioral changes, and moderate dehydration were noted, along with alternating sensorium with a tendency to sleep, unprovoked laughter, stasis, exhaustible clonus, and ataxia,^37^ adynamia, vertigo, urinary/fecal retention, gait disturbance.^35^**Neurological manifestations:** Neck stiffness, temporospatial disorientation, photophobia, confusion, altered language (palilalia, paraphasia, and aphasia), exaggerated reflexes,^37^,^38^ sensory depression, tremors and change in consciousness level.^46^**Complications:**- Opsoclonus-myoclonus-ataxia syndrome with gait instability, generalized myoclonus, tremor, and abnormal saccadic eye movements after resolution of fever.^36^	MRI in the brain and spinal cord.^35^,^37^*Serological testing*: MAC ELISA (IgM) in serum and/or cerebrospinal fluid,^35^,^38^ IgM-IFA, HI.^46^*Neutralization assay*: PRNT.^38^,^39^*Molecular testing*: RT-qPCR targeting the 5′UTR region and multiplex nested RT-PCR targeting the NS5 gene.^39^
ROCV	Reported case: fever, myalgia, arthralgia in both patients; one patient presented with prostration, abdominal pain, diarrhea, and thrombocytopenia, and the other had headache, retro-orbital pain, pruritus, nausea, and leukopenia.^40^	*Molecular testing*: RT-qPCR targeting E and NS5 genes,^30^ and nested PCR targeting the NS5 gene.^40^
MADV	Reported case: abrupt high-grade fever (39°C–40°C), asthenia, malaise, headache, nausea, and an intensely pruritic maculopapular rash; no arthralgia or myalgia.^43^	*Molecular testing*: RT-qPCR.^43^PRNT80.^44^*Serological testing*: IgG captures ELISA.^44^

### Comparative synthesis of clinical and diagnostic findings

The infections caused by the five arboviruses evaluated shared clinical manifestations such as fever, headache, myalgia, arthralgia, and retro-orbital pain. However, some distinctive characteristics were identified among the viruses.

In OROV infections, headache was reported as moderate to severe^6^,^7^ and was frequently accompanied by nausea, vomiting, abdominal pain,^11^,^12^ chills, or rash; meanwhile, MAYV infections were primarily characterized by persistent arthralgia,^22^ which in some cases lasted for several weeks.^30^ Furthermore, although both viruses shared the presence of a rash, this manifestation was described more frequently in MAYV, where in some cases it presented as a maculopapular rash.^22^,^47^

By contrast, SLEV infections presented a clearly distinct clinical profile, characterized by neurological manifestations^35^,^37^,^38^ such as spatial and temporal disorientation, tremor (general and upper limbs), malaise, ataxia, dysarthria, gait disturbances, language impairment, and behavioral changes, findings not reported for the other arboviruses included in this review.

By contrast, the single reported case of MADV presented a predominantly systemic and dermatological picture, notably an intensely pruritic maculopapular rash,^43^ unlike the exanthems described in MAYV. On the other hand, although the available evidence for ROCV was limited, the reported cases suggested the presence of abdominal pain, diarrhea, and hematological abnormalities, such as thrombocytopenia and leukopenia, in addition to symptoms shared with other arboviruses, such as myalgia and arthralgia.^41^

Overall, the comparative analysis of the arboviruses included in this review highlights both shared and distinct clinical and diagnostic features that must be interpreted in an integrated manner. While OROV and MAYV predominantly cluster within a febrile-musculoskeletal syndrome, SLEV is characterized by a neuroinvasive profile, and MADV and ROCV exhibit distinct systemic manifestations. However, these clinically recognizable patterns are often blurred in real-world settings due to the substantial overlap in early symptomatology and the limitations of routine diagnostic approaches.

In particular, serological cross-reactivity among co-circulating arboviruses represents a major constraint in accurate case classification. This phenomenon is evident in assays such as ELISA and immunofluorescence, which frequently detect cross-reactive antibodies between viruses such as MAYV and CHIKV^22^,^31^,^47^ or SLEV and DENV, potentially leading to misclassification. By contrast, more specific methods such as PRNT or molecular tests reduce this overlap and allow for clearer distinction between infections (Table 2). These findings highlight that, despite similar clinical presentations and shared diagnostic tools, antibody cross-reactivity remains a key challenge for accurate interpretation in endemic settings.

**Table 2:** Cross-reactivity patterns in serological assays among arboviruses.

Country	Infecting virus	Cross-reactive virus	Serological and molecular findings
Suriname^22^	MAYV	CHIKV	Immunofluorescence detected CHIKV IgG titers >64 in all samples (2/2) and IgM positivity in one case (1/2), despite negative CHIKV PCR results (2/2).
Argentina^38^	SLEV	DENV	A commercial DENV ELISA yielded IgM-positive results in all tested samples (9/9), indicating false-positive reactivity.
Brazil^39^	SLEV	DENV-4, YFV	Cross-neutralization assays showed partial neutralization with DENV-4 and YFV. Dengue RT-PCR results were negative across samples.
Multicountry^47^	MAYV	CHIKV	ELISA demonstrated cross-reactivity, with MAYV antibodies detected by CHIKV-targeted assays. Neutralization assays revealed limited cross-neutralization between MAYV and CHIKV.
Brazil and Peru^31^	MAYV	CHIKV	High cross-reactivity in ELISA (∼50% false positives overall); IgM cross-reactivity ∼64.7%; IgG ∼38.1% (asymmetric: higher in MAYV sera, 57.9%).

### Clinical impact of neglected arboviruses

Finally, some relevant clinical and diagnostic findings reported in the included studies are highlighted. These findings illustrate the clinical spectrum and severity associated with neglected arboviruses, ranging from neurological complications in SLEV infections^38^ to the high sensitivity of emerging diagnostic tools for detecting OROV.^18^ Furthermore, serious outcomes in vulnerable populations are underscored, including evidence of vertical transmission of OROV and associated adverse fetal and neonatal outcomes.^15^ Taken together, these observations provide a more comprehensive understanding of the clinical impact and public health relevance of these viruses, beyond the diagnostic challenges (Table 3).

**Table 3:** Summary of key findings from included studies.

Country	Population	Virus	Outstanding findings	Reference
Argentina	13 patients (mean of 38 years old)	SLEV	Some patients developed central nervous system involvement, including meningitis and encephalitis. Electroencephalography revealed non-convulsive epileptiform activity in the temporal region and diffuse slow-wave abnormalities.	Seijo, A, *et al*. 2011
Brazil	1 patient (55-year-old male)	SLEV	MRI revealed multiple inflammatory lesions in brain white matter and cervical/thoracic spinal cord. The patient also depicted myelin thinning in the same topography of the corresponding lesions.	Pedrosa, DA, *et al*. 2024
Brazil	6 pregnant women	OROV	Pregnancy outcomes included one miscarriage, three intrauterine fetal demises, and one case without fetal loss. Additionally, detection of OROV RNA in multiple fetal tissues including brain, kidney, lung, heart, spleen, placenta, and umbilical cord, indicating possible vertical transmission.	Medeiros DBA, *et al*. 2025
Brazil	3 neonates born to mothers with OROV infection	OROV	Fetal/neonatal: severe microcephaly, craniofacial disproportion, arthrogryposis, hydrops fetalis, hypotonia, absence of reflexes, feeding difficulties, seizures, developmental delay, ventriculomegaly, and camptodactyly of the hand.	Ribeiro BFR, *et al*. 2025

## Discussion

Although some South American countries have reported an increase in the circulation of neglected arboviruses such as OROV and MAYV in recent years,^48–50^ surveillance and continuous monitoring of these viruses remain insufficient, especially in tropical, rural, and remote areas. Therefore, to be prepared for possible future outbreaks caused by these arboviruses, this review aims to provide an overview of the main clinical manifestations and complications caused by OROV, MAYV, SLEV, ROCV, and MADV, as well as the various diagnostic techniques used in South America for their detection.

It has been shown that one of the main obstacles is the risk of an increase in misdiagnoses, because several endemic arboviruses (dengue, Zika, and CHIKV) present with similar clinical manifestations, making differential diagnosis difficult.^51^ For example, most acute undifferentiated febrile illnesses in the Americas are attributed to dengue infections; however, only one-third of cases clinically diagnosed as dengue are caused by this virus, leaving co-endemic or emerging diseases undetected.^52^ This especially occurs in settings where diagnostic resources are limited, and in some cases, the causative agents remain undiagnosed across most of the Amazon region.^53^

According to the findings of this review, OROV cases have been reported in countries such as Brazil, Bolivia, Colombia, Ecuador, Paraguay, and Peru,^6^,^9^,^12^,^14^ indicating an expansion in the geographic distribution of this virus. This is supported by the findings of some authors, who have documented multiple independent introductions into Colombia from Ecuador and Peru.^13^ In addition, the wide variety of reported clinical manifestations remains non-specific and like those of other febrile illnesses.

Among the most concerning findings regarding OROV infections is evidence of possible vertical transmission.^15^ The detection of viral RNA in multiple fetal tissues, including the central nervous system, reinforces the virus’s neurotropic potential and its ability to cross the placental barrier. These findings show similarities to infections caused by the Zika virus, where abnormal brain development, microcephaly, and arthrosis have been observed in surviving newborns.^54^ Although the number of reported cases is limited, the potential adverse outcomes are a cause for concern. Therefore, it is essential to conduct additional studies to characterize these mechanisms of vertical transmission.

On the other hand, findings regarding MAYV infection have also revealed a geographic expansion of the virus in South America. In countries such as Brazil and Colombia, phylogenetic analyses have identified genotype D, which is closely related to strains previously reported in Peru,^28^,^29^ suggesting sustained circulation of this lineage in the region. Furthermore, a worrying finding is the specificity of diagnostic methods for detecting this virus, as serological and immunofluorescence tests have shown cross-reactivity with viruses such as CHIKV,^22^,^31^,^47^ hindering accurate case confirmation.

In the case of SLEV infections, clinical manifestations have demonstrated neuroinvasive potential, which underscores the need to include the aforementioned arboviruses in syndromic surveillance of viral encephalitis. However, this has not been taken into account in either health policies or daily clinical practice in the region.^17^,^38^,^42^ The wide diversity of neurological manifestations, ranging from meningitis and encephalitis to language disorders, altered mental status, and syndromes such as opsoclonus-myoclonus-ataxia,^36^ highlights the clinical complexity of the virus and its potential to generate severe conditions that may go unnoticed or be attributed to other viral etiologies.

Although most cases of SLEV have been reported in the USA, the virus’s distribution has spread from Canada to Argentina,^55^ suggesting an underestimated risk in Latin America, especially in areas where other flaviviruses co-circulate. In this context, diagnosis presents a significant challenge, as widely used serological tests such as MAC-ELISA can cross-react with other viruses of the same serocomplex or family, such as dengue,^38^ limiting their specificity. While confirmatory techniques such as PRNT and molecular methods (RT-qPCR) improve diagnostic accuracy, their availability is limited in resource-constrained settings, which could contribute to underdiagnosis and an incomplete understanding of the true burden of SLEV disease in the region.

In the case of ROCV, gastrointestinal symptoms and hematological abnormalities were observed,^40^ while MADV infections were characterized by high fever and pruritic rash.^43^ However, previous literature indicates that both viruses are neurotropic and have been associated with neurological manifestations, although these clinical presentations were not reported in the studies included in this review.^43^,^56^ Although numerous studies in humans have not been conducted for either virus, their circulation continues to be reported in the region. For example, the presence of MADV in *Culex* mosquitoes was reported in Argentina,^57^ and in Brazil, after four decades, the circulation of ROCV in horses was reported.^56^ Therefore, based on these findings, it is important to consider strengthening epidemiological surveillance networks and implementing integrated vector prevention and control strategies, including reducing breeding sites, using insecticide-treated bed nets and residual spraying.^58^

Currently, epidemiological surveillance systems play a fundamental role in monitoring arbovirus transmission and in the early detection of outbreaks;^2^ however, the evidence suggests that neglected and emerging arboviruses continue to be under-reported. Furthermore, although diagnostic algorithms and guidelines have been developed for some of these viruses, such as MAYV and OROV,, ^59^ diagnostic capacity remains insufficient, especially at the primary and secondary levels of care. These limitations can delay case confirmation, hinder the timely detection of outbreaks, and compromise the early implementation of response measures.

Finally, this review has some limitations that should be considered when interpreting the findings. First, the search was restricted to studies published from January 2005 to March 2026 to capture current epidemiological patterns; therefore, relevant studies published before January 2005 may have been excluded. Second, most of the included studies are from Brazil and Peru, which may limit the generalizability of the clinical and diagnostic findings to other countries. Third, a limited number of studies were identified for ROCV and MADV, and much of the available evidence on these arboviruses was derived from case reports and small observational studies; while these studies provided valuable information, their limited sample size may reduce the external validity of the reported findings.

## Conclusion

The presence of neglected arboviruses has been confirmed in South America, posing an emerging threat, particularly in rural, tropical, and resource-poor areas. It has been shown that the clinical manifestations are very similar to those of better-known arboviruses, which can lead to diagnostic errors and contribute to an increase in potential outbreaks. Furthermore, given the diversity of circulating arboviruses and the potential for serological cross-reactivity that emerging viruses may cause, diagnostic results must be interpreted with caution. Although molecular detection methods currently offer high sensitivity and specificity, their implementation in remote and underserved settings remains a challenge. On the other hand, infrastructure limitations, restricted access to diagnostic tools, and insufficient knowledge among healthcare personnel, continue to hinder timely and accurate diagnosis and surveillance. In this context, it is important to adopt a preventive and comprehensive approach that contributes to the implementation of appropriate diagnostic techniques and the ongoing training of healthcare personnel, with the aim of controlling the spread of these viruses, detecting potential outbreaks early, and reducing the impact on the population.

## Supplementary Material

trag095_Supplemental_Files

## Data Availability

The data supporting the findings of this review are available within the article and the cited references.
