# Supplementary material for: Neglected arboviruses in South America: clinical manifestations and diagnostic challenges
Source: Trans R Soc Trop Med Hyg. 2026 Aug 27;120(11):1096–106. doi: 10.1093/trstmh/trag095 (PMC13728643; doi:10.1093/trstmh/trag095)
Supplement: Supplementary file 1 [file trag095_supplemental_files.zip › Supplementary material. Figures and tables.docx]

**Supplementary Figure 1. Study selection flow chart according to the PRISMA guide.**

**Screening**

**Identification**

**Identification of studies via others methods**

**Identification of studies via databases and registers**

Records identified from:

Pubmed (n = 173)

Scopus (n = 40)

LILACS (n = 51)

Google scholar (n = 107)

Total (n = 371)

Records removed *before screening*:

Duplicate records removed (n = 19)

Hand searching

(n = 6)

Records excluded:

Wrong outcome: (n = 258)

Wrong study design: (n = 36)

Wrong population: (n = 21)

Wrong publication type: (n = 2)

Duplicated (n = 3)

Total: (n = 320)

Records screened based on title/abstract (n = 352)

Reports assessed for eligibility (n = 32)

Reports of included studies

**(n = 38)**

**Included**

**Supplementary Table 1. Literature Search Strategy**

**Box S1: Search strategy in PubMed (173)**

| #1 | ("Arbovirus Infections"[Mesh] OR arbovirus*[tiab] OR mayaro virus[tiab] OR mayaro virus[Mesh] OR oropouche virus[tiab] OR oropouche virus[Mesh] OR "Rocio virus"[tiab] OR "Saint Louis encephalitis virus"[tiab] OR "St. Louis encephalitis"[tiab] OR "Madariaga virus"[tiab] OR “Eastern Equine Encephalitis Virus”[tiab]) |
| --- | --- |
| #2 | ("Signs and Symptoms"[Mesh] OR "Clinical Manifestations"[tiab] OR "Symptomatology"[tiab] OR "Symptoms"[tiab] OR "Signs"[tiab] OR "clinical presentation"[tiab] OR "clinical features"[tiab]) |
| #3 | ("Diagnosis"[Mesh] OR "diagnostic techniques and procedures"[Mesh] OR diagnos*[tiab] OR "molecular test*"[tiab] OR "serological test*"[tiab] OR "point of care"[tiab] OR "ELISA"[tiab] OR "RT-PCR"[tiab] OR "rapid diagnostic"[tiab]) |
| #4 | ("South America"[Mesh] OR "South America"[tiab] OR "Latin America"[tiab] OR Sudamérica[tiab] OR Argentina[tiab] OR Bolivia[tiab] OR Brazil[tiab] OR Chile[tiab] OR Colombia[tiab] OR Ecuador[tiab] OR Guyana[tiab] OR Paraguay[tiab] OR Peru[tiab] OR Suriname[tiab] OR Uruguay[tiab] OR Venezuela[tiab] OR "French Guiana"[tiab]) |
| #5 | #1 AND # 2 AND #3 AND #4 |
| #6 | ("Arbovirus Infections"[Mesh] OR arbovirus*[tiab] OR mayaro virus[tiab] OR mayaro virus[Mesh] OR oropouche virus[tiab] OR oropouche virus[Mesh] OR "Rocio virus"[tiab] OR "Saint Louis encephalitis virus"[tiab] OR "St. Louis encephalitis"[tiab] OR "Madariaga virus"[tiab] OR "Encephalitis Virus, Eastern Equine"[Mesh]) AND ("Signs and Symptoms"[Mesh] OR "Clinical Manifestations"[tiab] OR "Symptomatology"[tiab] OR "Symptoms"[tiab] OR "Signs"[tiab] OR "clinical presentation"[tiab] OR "clinical features"[tiab]) AND ("Diagnosis"[Mesh] OR "diagnostic techniques and procedures"[Mesh] OR diagnos*[tiab] OR "molecular test*"[tiab] OR "serological test*"[tiab] OR "point of care"[tiab] OR "ELISA"[tiab] OR "RT-PCR"[tiab] OR "rapid diagnostic"[tiab]) AND ("South America"[Mesh] OR "South America"[tiab] OR "Latin America"[tiab] OR Sudamerica[tiab] OR Argentina[tiab] OR Bolivia[tiab] OR Brazil[tiab] OR Chile[tiab] OR Colombia[tiab] OR Ecuador[tiab] OR Guyana[tiab] OR Paraguay[tiab] OR Peru[tiab] OR Suriname[tiab] OR Uruguay[tiab] OR Venezuela[tiab] OR "French Guiana"[tiab]) |
| Filters | Filters applied: Abstract, Case Reports, Clinical Study, Comparative Study, Evaluation Study, Multicenter Study, Observational Study, Validation Study, English, Portuguese, Spanish, Humans, from 2005/1/1 - 2026/02/28 |

**Box S2: Search strategy Scopus (40)**

| #1 | (TITLE-ABS-KEY("mayaro virus" OR "oropouche virus" OR "rocio virus" OR "saint louis encephalitis virus" OR "st. louis encephalitis" OR "madariaga virus" OR "Eastern Equine Encephalitis Virus")) |
| --- | --- |
| #2 | (TITLE-ABS-KEY("signs and symptoms" OR "clinical manifestations" OR "symptomatology" OR "symptoms" OR "signs" OR "clinical presentation" OR "clinical features")) |
| #3 | (TITLE-ABS-KEY("diagnosis" OR "diagnostic techniques" OR "molecular test" OR "serological test" OR "point of care" OR "ELISA" OR "RT-PCR" OR "rapid diagnostic")) |
| #4 | (TITLE-ABS-KEY("south america" OR "latin america" OR "sudamerica" OR "argentina" OR "brazil" OR "peru" OR "venezuela")) |
| #5 | #1 AND # 2 AND #3 #4 |
| #6 | ( TITLE-ABS-KEY ( "mayaro virus" OR "oropouche virus" OR "rocio virus" OR "saint louis encephalitis virus" OR "st. louis encephalitis" OR "madariaga virus" OR "Eastern Equine Encephalitis Virus")) AND ( TITLE-ABS-KEY ( "signs and symptoms" OR "clinical manifestations" OR "symptomatology" OR "symptoms" OR "signs" OR "clinical presentation" OR "clinical features" ) ) AND (TITLE-ABS-KEY ( "diagnosis" OR "diagnostic techniques" OR "molecular test" OR "serological test" OR "point of care" OR "ELISA" OR "RT-PCR" OR "rapid diagnostic" ) ) AND ( TITLE-ABS-KEY ( "south america" OR "latin america" OR "sudamerica" OR "argentina" OR "brazil" OR "peru" OR "venezuela" OR "colombia" OR "paraguay" OR "ecuador" OR "chile" OR "uruguay" OR "guayana" OR "suriname" OR "french guayana" )) |
| Filters | Medicine, Immunology and Microbiology, Biochemistry, Genetics, and Molecular Biology, Neuroscience, article, book chapter, South America, spanish, portuguese, english, 2005/1/1 - 2026/02/28. |

**Box S3: Search strategy in LILACS (51)**

| #1 | (Arbovirus Infections OR arbovirus* OR "mayaro virus" OR "oropouche virus" OR "rocio virus" OR "Saint Louis encephalitis virus" OR "St. Louis encephalitis" OR "Madariaga virus" OR “Eastern Equine Encephalitis Virus”) |
| --- | --- |
| #2 | ("Signs and Symptoms" OR "Clinical Manifestations" OR Symptomatology OR Symptoms OR Signs OR "clinical presentation" OR "clinical features") |
| #3 | (Diagnosis OR "diagnostic techniques and procedures" OR diagnos* OR "molecular test*" OR "serological test*" OR "point of care" OR ELISA OR RT-PCR OR "rapid diagnostic") |
| #4 | ("South America" OR "Latin America" OR Sudamérica OR Argentina OR Bolivia OR Brazil OR Chile OR Colombia OR Ecuador OR Guyana OR Paraguay OR Peru OR Suriname OR Uruguay OR Venezuela OR "French Guiana") |
| #5 | #1 AND # 2 AND #3 AND #4 |
| #6 | (Arbovirus Infections OR arbovirus* OR "mayaro virus" OR "oropouche virus" OR "rocio virus" OR "Saint Louis encephalitis virus" OR "St. Louis encephalitis" OR "Madariaga virus" OR “Eastern Equine Encephalitis Virus”) AND ("Signs and Symptoms" OR "Clinical Manifestations" OR Symptomatology OR Symptoms OR Signs OR "clinical presentation" OR "clinical features") AND (Diagnosis OR "diagnostic techniques and procedures" OR diagnosis* OR "molecular test*" OR "serological test*" OR "point of care" OR ELISA OR RT-PCR OR "rapid diagnostic") AND ("South America" OR "Latin America" OR Sudamérica OR Argentina OR Bolivia OR Brazil OR Chile OR Colombia OR Ecuador OR Guyana OR Paraguay OR Peru OR Suriname OR Uruguay OR Venezuela OR "French Guiana") |
| Filters | Filters applied: estudio diagnóstico, estudio observacional, factores de riesgo, estudio de tamizaje, estudio de prevalencia, guía de práctica clínica, estudio de evaluación, estudio de etiología, spanish, portuguese, english, 2005/1/1 - 2026/02/28. |

**Box S2: Search strategy Google Scholar (107)**

| #1 | ("mayaro virus" OR "oropouche virus" OR "rocio virus" OR "saint louis encephalitis virus" OR "st. louis encephalitis" OR "madariaga virus" OR “eastern equine encephalitis virus”) |
| --- | --- |
| #2 | ("signs and symptoms" OR "clinical manifestations" OR symptomatology OR symptoms OR signs OR "clinical presentation" OR "clinical features") |
| #3 | ("diagnosis" OR "diagnostic techniques and procedures" OR "molecular test" OR "serological test" OR "point of care" OR ELISA OR RT-PCR OR "rapid diagnostic") |
| #4 | ("south america" OR "latin america" OR sudamérica OR argentina OR bolivia OR brazil OR chile OR colombia OR ecuador OR guyana OR paraguay OR peru OR suriname OR uruguay OR venezuela OR "french guiana") |
| #5 | #1 AND # 2 AND #3 AND #4 |
| #6 | ("mayaro virus" OR "oropouche virus" OR "rocio virus" OR "saint louis encephalitis virus" OR "st. louis encephalitis" OR "madariaga virus" OR “eastern equine encephalitis virus”) AND ("signs and symptoms" OR "clinical manifestations" OR symptomatology OR symptoms OR signs OR "clinical presentation" OR "clinical features") AND ("diagnosis" OR "diagnostic techniques and procedures" OR "molecular test" OR "serological test" OR "point of care" OR ELISA OR RT-PCR OR "rapid diagnostic") AND ("south america" OR "latin america" OR sudamérica OR argentina OR bolivia OR brazil OR chile OR colombia OR ecuador OR guyana OR paraguay OR peru OR suriname OR uruguay OR venezuela OR "french guiana") |
| Filters | Filters applied: spanish, 2005 - 2026. |

**Supplementary Table 2**. Risk of bias assessment of the included studies using the Joanna Briggs Institute (JBI) critical appraisal tools. (see Excell atach).

**Supplementary Table 3.** Characteristics of the 38 studies included in the review, including country, subnational location, study population, and neglected arbovirus investigated.

| **N°** | **Authors / Year of Publication** | **Country** | **Subnational Level** | **Population** | **Virus** |
| --- | --- | --- | --- | --- | --- |
| 1 | Spinsanti, LI, et al., 2008 | Argentina | Cordoba | Among 72 tested samples, 47 (65.2%) were confirmed cases of SLEV infection. | SLEV |
| 2 | Seijo, A. et al. 2011 | Argentina | Buenos Aires | Thirteen patients with OROV infection [mean age: 38 years]. | SLEV |
| 3 | Carballo, C., et al., 2016 | Argentina | Buenos Aires | One 8-year-old female patient. | SLEV |
| 4 | Saguier Padilla I, et al., 2025 | Argentina | Buenos Aires | One 35-year-old male patient. | SLEV |
| 5 | Escalera-Antezana, JP., et al. 2025 | Bolivia | Beni | Among 537 tested samples, 76 (14.2%) were confirmed cases of OROV infection. | OROV |
| 6 | Coimbra TL., et al., 2007 | Brazil | Mato Grosso | Three male patients [aged 36, 74, and 79 years presenting acute febrile illness]. | MAYV |
| 7 | Coimbra TLM., et al., 2008 | Brazil | Sao Paulo | One ROCV isolate was obtained from a human brain specimen. | ROCV |
| 8 | Cruz, AC., et al., 2009 | Brazil | Pará | Among 1,597 human serum samples analyzed, 90 (5.6%) were positive for OROV, 20 (1.3%) for MAYV, and 6 (0.4%) for SLEV. | OROV / MAYV / SLEV |
| 9 | Barbosa Costa G, et al., 2019 | Brazil | Minas Gerais | Among 240 serum samples tested, 11 (4.6%) were positive for SLEV. | SLEV |
| 10 | Romeiro MF, et al., 2020 | Brazil | Sao Paulo | MAYV seropositivity was detected in 47 of 5,608 asymptomatic blood donors (0.8%). | MAYV |
| 11 | Saivish MV., et al., 2020 | Brazil | Goias | Among 647 tested serum samples, 02 (0.3%) were confirmed cases of ROCV infections. | ROCV |
| 12 | de Paula Silveira-Lacerda, et al., 2021 | Brazil | Goias | Among 375 individuals tested, 26 (6.9%) were confirmed to have MAYV infection. | MAYV |
| 13 | Saatkamp CJ, et al., 2021 | Brazil | Pará | Among 49 patients with acute febrile illness, 4 (8.2%) were confirmed as MAYV-positive. | MAYV |
| 14 | de Lima RC, et al., 2024 | Brazil | Amapá | Among 166 febrile patients tested, 17 (10.2%) were confirmed to have OROV infection. | OROV |
| 15 | Pimentel, EP., et al., 2024 | Brazil | Rondonia | Among 857 individuals tested, one (0.1%) was confirmed to have MAYV infection. | MAYV |
| 16 | Moreira, HM. et al., 2024 | Brazil | Amazonas, Rondonia | Among 351 patients with acute febrile illness, 27 (7.7%) were OROV-positive (17 from Amazonas and 10 from Rondônia). | OROV |
| 17 | Pedrosa, DA., et al., 2024 | Brazil | Amazonas | One 55-year-old male patient. | SLEV |
| 18 | Gomes Mourão, MP., et al., 2025 | Brazil | Amazonas | Among 644 serum samples, 51 (7.9%) were OROV-positive, including 5 OROV–DENV coinfections. | OROV |
| 19 | Medeiros DBA, et al., 2025 | Brazil | Pernambuco | Six pregnant women with OROV infection. | OROV |
| 20 | Ribeiro BFR, et al., 2025 | Brazil | Acre | Three neonates born to mothers with confirmed OROV infection during pregnancy. | OROV |
| 21 | Ciuoderis KA., et al., 2022 | Colombia | Amazonas, Norte de Santander, Valle del Cauca, and Meta | Among 2,967 patients with febrile syndrome, 105 (3.5%) OROV infections were identified, including cases from Amazonas (n=48), Meta (n=51), Valle del Cauca (n=3), and Norte de Santander (n=3). | OROV |
| 22 | Grisales-Nieto JC, et al., 2024 | Colombia | Amazonas | One 63-year-old female patient. | OROV |
| 23 | Sánchez-Lerma, L., et al., 2024 | Colombia | Meta | Among 100 patients with acute febrile illness, 27 (27.0%) were MAYV-positive. | MAYV |
| 24 | Perez-Restrepo LS, et al., 2024 | Colombia | Amazonas | MAYV infection was confirmed in 2 of 1,460 patients with acute febrile illness (0.1%). | MAYV |
| 25 | Rivera Mora L., et al., 2025 | Ecuador | Bolivar | One 62-year-old male patient. | OROV |
| 26 | Rojas A, et al., 2021 | Paraguay | Asuncion | Among 100 patients with acute febrile syndrome, no OROV infections were detected. | OROV |
| 27 | Alvarez-Falconi, P., & Rios Ruiz, B. 2010 | Peru | San Martin | Among 171 patients with acute febrile illness, 108 (63.2%) were OROV-positive. | OROV |
| 28 | Halsey ES, 2013 | Peru | Loreto, Junín, Madre de Dios | MAYV infection was confirmed in 16 of 2,094 febrile participants (0.8%) | MAYV |
| 29 | Castro, S. et al., 2013 | Peru | Cajamarca | Among 33 patients with suspected dengue, 17 of 26 tested (65.4%) were OROV-positive. | OROV |
| 30 | Alva-Urcia C, et al., 2017 | Peru | Madre de Dios | Among 139 patients with AFI, 12 (8.6%) were OROV-positive. | OROV |
| 31 | Huancas de la Cruz C., et al., 2019 | Peru | Amazonas | One 35-year-old female patient. | MAYV |
| 32 | Silva-Caso, W. et al., 2019 | Peru | Huánuco | Among 268 individuals, 46 (17.2%) patients were OROV-positive. | OROV |
| 33 | Pérez JG, et al., 2019 | Peru | Loreto | MAYV infection was confirmed in 17 of 70 participants (24.3%), while MADV infection was confirmed in 1 participant (1.4%). | MAYV / MADV |
| 34 | Aguilar-Luis, MA et al., 2020 | Peru | Cajamarca | Among 359 patients with suspected febrile illness, 40 (11.1%) were MAYV-positive. | MAYV |
| 35 | Hassing RJ., et al., 2010 | Suriname | Non specified | Two Dutch travelers infected with MAYV [48-year-old]. | MAYV |
| 36 | Blohm GM., et al., 2018 | Venezuela | Lara | One 12-year-old patient. | MADV |
| 37 | Henss L, et al., 2019 | Brazil / Bolivia | Rio de Janeiro / Beni | Clinical samples positive for MAYV (Bolivia), and CHIKV (Brazil). | MAYV |
| 38 | Fischer C, et al., 2020 | Peru / Brazil | Loreto, Piura, Lambayeque, Cusco, and Junín / Rio de Janeiro and Bahia | 101 serum samples, including 37 MAYV-positive samples from Peru and 64 CHIKV-positive samples from Brazil. | MAYV |
